# Molecular Basis of Class B GPCR Selectivity for the Neuropeptides PACAP and VIP

**DOI:** 10.3389/fmolb.2021.644644

**Published:** 2021-03-25

**Authors:** Chenyi Liao, Jacob M. Remington, Victor May, Jianing Li

**Affiliations:** ^1^ Department of Chemistry, University of Vermont, Burlington, VT, United States; ^2^ State Key Laboratory of Molecular Reaction Dynamics, Dalian Institute of Chemical Physics, Dalian, China; ^3^ Department of Neuroscience, University of Vermont, Burlington, VT, United States

**Keywords:** G protein-coupled receptor, membrane protein, signaling, molecular dynamics, conformational transition, ligand selectivity

## Abstract

The related neuropeptides PACAP and VIP, and their shared PAC1, VPAC1 and VPAC2 receptors, regulate a large array of physiological activities in the central and peripheral nervous systems. However, the lack of comparative and molecular mechanistic investigations hinder further understanding of their preferred binding selectivity and function. PACAP and VIP have comparable affinity at the VPAC1 and VPAC2 receptor, but PACAP is 400–1,000 fold more potent than VIP at the PAC1 receptor. A molecular understanding of the differing neuropeptide-receptor interactions and the details underlying the receptor transitions leading to receptor activation are much needed for the rational design of selective ligands. To these ends, we have combined structural information and advanced simulation techniques to study PACAP/VIP binding selectivity, full-length receptor conformation ensembles and transitions of the PACAP/VIP receptor variants and subtypes, and a few key interactions in the orthosteric-binding pocket. Our results reveal differential peptide-receptor interactions (at the atomistic detail) important for PAC1, VPAC1 and VPAC2 receptor ligand selectivity. Using microsecond-long molecular dynamics simulations and the Markov State Models, we have also identified diverse receptor conformational ensembles and microstate transition paths for each receptor, the potential mechanisms underlying receptor open and closed states, and the interactions and dynamics at the transmembrane orthosteric pocket for receptor activation. These analyses reveal important features in class B GPCR structure-dynamics-function relationships, which provide novel insights for structure-based drug discovery.

## Introduction

Pituitary adenylate cyclase-activating peptide (PACAP, *ADCYAP1*, P18509) and vasoactive intestinal polypeptide (VIP, *VIP*, P01282) are two important neuropeptides for neural development, body calcium homeostasis, glucose metabolism, circadian rhythm, thermoregulation, inflammation, feeding behavior, pain modulation, stress and related endocrine response (Harmar et al., 2012; Bortolato et al., 2014; Culhane et al., 2015; Liao et al., 2019b; Liao et al., 2019c). Their important roles are mediated by activating three class B G protein-coupled receptors (GPCRs) including the PAC1 (*ADCYAP1R1* and related splice variants), VPAC1 (*VIPR1)*, and VPAC2 (*VIPR2)* receptors, which share over 60% sequence similarity within this GPCR subtype. Interestingly, PACAP and VIP display distinct selectivity for the PAC1 and VPAC1/2 receptors (Vaudry et al., 2009): PACAP (PACAP38 and the C-terminally truncated PACAP27) and VIP have comparable high affinity for the VPAC1 and VPAC2 receptors (Gottschall et al., 1990; Lam et al., 1990), whereas the PACAP peptides are 400–1,000 fold more potent than VIP as agonists for PAC1 receptor (Gottschall et al., 1990; Dautzenberg et al., 1999).

From recent progress in X-ray crystallography and cryogenic electron microscopy (cryo-EM) (Hollenstein et al., 2013; Yang et al., 2015; Jazayeri et al., 2017; Zhang et al., 2017a; Zhang et al., 2017b; Zhang et al., 2018; Duan et al., 2020; Kobayashi et al., 2020; Liang et al., 2020; Wang et al., 2020), several class B receptor structures have been established. We have now integrated the structural information with simulations, theory and available experimental findings to examine: *1)* differences in intrinsic PAC1 and VPAC1/2 receptor dynamics; *2)* differential PACAP and VIP interactions and energetic impacts at the receptor subtypes; and *3)* the interactions and dynamical features of PACAP-induced activation of the PAC1null receptor. Class B GPCRs have been suggested to have receptor features and dynamics distinct from those described for class A receptors, presumably because of the presence of the extracellular domain (ECD) (Liao et al., 2017), the apparent absence of “toggle switches” (Li et al., 2013), and other signature features. The class B receptors are likely to adopt unique conformational changes in the heptahelical transmembrane (7TM) domain following neuropeptide binding for receptor activation. Different from some class B receptors (Hollenstein et al., 2014; Yang et al., 2015; Graaf et al., 2016; Jazayeri et al., 2016; Jazayeri et al., 2017; Song et al., 2017; Zhang et al., 2017a; Zhang et al., 2017b; Liang et al., 2018), the detailed and comparative examination of the PAC1 and VPAC1/2 receptor function and actions have not been well investigated. Accordingly, we have integrated long-timescale molecular dynamics (MD) simulations with other computational and theoretical techniques to study the conformational ensembles and transitions of two PAC1 receptor variants (namely PAC1null and PAC1s) (Liao et al., 2019c), VPAC1, and VPAC2 receptors, as well as their interactions with the neuropeptides PACAP and VIP. The PAC1null receptor has no intracellular loop 3 (ICL3) inserts (neither hip nor hop inserts) from alternative splicing, and represents one of the most prevalent receptor isoforms in the central nervous system (CNS). The PAC1s receptor is similar to the PAC1null variants, but has a 21-amino acid deletion (residues 89–109) in the ECD. For reasons unknown, the PAC1s receptor is not highly expressed in the CNS (or any tissue) and yet this variant was chosen for structural determination (Sun et al., 2007; Kumar et al., 2011; Wang et al., 2020). Distinct from structural determination, our simulations and analyses detail the receptor conformational transitions and peptide interactions that are biologically relevant to the receptor subtype. While the PAC1 receptor has been recognized as an emerging target for stress-related disorders (Ressler et al., 2011), our studies, for the first time, reveal the molecular basis underlying PACAP receptor selectivity which may be essential for the rational design of effective therapeutic agents.

## Models and Methods

### Model Preparation and MD Simulation Setup

The ligand-free and ligand-binding full-length receptor systems. Our homology modeling was carried out with the protein modeling program *Prime* (Schrödinger, *Inc.*) (Zhao et al., 2011; Li et al., 2011) which built the full-length VPAC1 and VPAC2 including the ECD, the 7TM, as well as the linker loop (see details in Supplementary Table S2). The PAC1s model was modified from our previous PAC1null receptor model (Liao et al., 2017) by deleting the 21-amino acid in ECD. For each receptor, we generated a number of initial models which were distinct in the ECD orientation to better sample different conformational states (Supplementary Figure S1). The Membrane Builder in CHARMM-GUI (Jo et al., 2008) was employed to build the protein/membrane complex systems, each of which contains a receptor with/without a ligand, a lipid bilayer of ∼ 225 POPC molecules, ∼ 27,000 TIP3P water molecules, counter ions, and 0.12 M NaCl, totaling ∼ 120,000 atoms in a periodic box ∼94 × 94 × 147Å^3^.

The ligand-ECD complex systems. To understand the distinct binding affinity of PACAP/VIP for PAC1null, PAC1s, VPAC1 and VPAC2 receptors, we simulated the ECD of each receptor and PACAP/VIP in a solvent environment. PACAP/VIP was initially placed near the position based on known peptide-binding modes to class B GCPRs (Sun et al., 2007; Grace et al., 2010; Parthier et al., 2007; Pioszak and Xu, 2008) (Supplementary Figure S2). We used the PDB Reader of CHARMM-GUI (Jo et al., 2008) to prepare protein then solvated and neutralized with addition of 0.12 M NaCl in VMD (Humphrey et al., 1996) (see our simulation summary in Supplementary Table S1).

Our simulations were performed with the CHARMM36-cmap force field (Best et al., 2012). Each system went through energy minimization, 285-ns multiple-step equilibrations, and MD simulations using the NAMD package (Phillips et al., 2005). Both equilibration and production runs were performed in the NPT ensemble (310K, 1 bar, Langevin thermostat and Nose-Hoover Langevin barostat) with a time step of 2 fs. All of the bond lengths to the hydrogen atoms in the protein or lipid molecules were constrained. The particle mesh Ewald (PME) technique was used for the electrostatic calculations. The van der Waals and short-range electrostatics were cut off at 12.0 Å with switch at 10.0 Å. Each system was carried out 1000 ns for 4–5 replicas.

Enhanced sampling for ligand-binding simulations. Adaptive tempering was employed to enhance conformational sampling under PACAP or PACAP6-38 insertion for ∼ 400 ns, in which the simulation temperature was dynamically updated as a continuously random variable in the range of 310–360 K with the Langevin equation (Zhang and Ma, 2010) implemented in NAMD (Supplementary Figure S3).

## Markov state model

Markov state models (MSMs) of molecular kinetics were applied on the collection of MD trajectories, from which we identified the transition pathways and estimated the long-time statistical dynamics between the conformational states of interest (Pande et al., 2010; Prinz et al., 2011). The ligand-free full-length receptor MD trajectories were used for MSM construction. We used the MSMBuilder 3.8.0 program Bowman et al., 2009a; Bowman et al., 2009b to construct the transition matrices of MSM. Trajectories that contain stable conformational states (measured by C_α_ root-mean-square deviation (RMSD) in Supplementary Figure SM2) were prepared by saving the C_α_ coordinates of a GPCR protein with atomic indices. Thus, we have 4,519–14,281 conformations in each trajectory. We grouped the conformational ensembles into a set of clusters, called microstates, based on C_α_ RMSD using the *k*-centers algorithm with 33–55 cluster centers Bowman et al., 2009b.

With the microstate discretization, we used the maximum likelihood estimation to build the reversible transition matrices at the lag time series. The evolution of the transition probability between closed and open conformational states of a receptor protein was evaluated at a series of lag times *τ*, 2*τ*, …, n*τ* by performing the *Chapman-Kolmogorov* test (Prinz et al., 2011) which confirmed that our model at the chosen lag time is Markovian (Chodera et al., 2007; Noé et al., 2009; Prinz et al., 2011) (Supplementary Figures SM4,SM5). Finally, this was achieved at a lag time of 1.2–2.4 ns as judged by flat lines in the *implied timescales* plots (Supplementary Figure SM3) (see Ref. Liao et al., 2019a for our detailed implementation of the *Chapman-Kolmogorov* test).

Using the transition-path theory (TPT) (Weinan and Vanden-Eijnden, 2006; Berezhkovskii et al., 2009; Metzner et al., 2009; Noé et al., 2009), we calculated the transition path connecting between the stable conformational states in a transition matrix and estimated the time quantity to travel from one set of states to the others by the lag time divided by the minimum net flux in the transition path (Supplementary Table S3). Thus, the transition time refers to the time quantity to complete one transition between two stable conformational states. The division of macrostates were calculated from the eigenfunction structure using the *Perron Cluster Cluster Analysis* (PCCA) method (Noé et al., 2007). Supplementary Figure SM6 illustrates the 3D projections of the microstates and macrostate divisions in accordance to ECD orientation and position by backbone center-of-mass (COM) distances between ECD and extracellular loop 1 (ECL1) (d_ECD-ECL1_) and extracellular loop 3 (ECL3) (d_ECD-ECL3_), respectively. Detailed constructions and validation of MSM were described in the Supplementary Material.

### Data Analysis

Binding free energy (ΔG_b_) of PACAP/VIP peptide bound to the ECD and per-residue free energy decomposition were computed over the last 500 ns MD trajectories using the Molecular Mechanics Generalized Born Surface Area (MM-GBSA) method (Miller et al., 2012) by MMPBSA.py tool (version: 14.0) in the Amber package (Case, 2018). The second modified Bondi radii set were used in the GB method. ΔG_b_ was given as an average of five replicas in each system. Conformational analysis and distance analysis were performed with TCL scripts implemented in VMD 1.9.1 (Humphrey et al., 1996) and plotted by matplotlib. The average distances involving a group of residues were calculated by the distance between the backbone COMs of groups of residues. Water density map were calculated using MDAnalysis python package. The tilt angle of ECD is defined as the angle between the vector along the N-terminal helix and the Z-axis. For example, the vector along the N-terminal helix of PAC1 ECD is calculated by summing the C-O vectors along the helical residues 30–47.

## Results and Discussion

### Conformation Ensembles and Transitions of PAC1 and VPAC1/2 Receptors

**FIGURE 1:**
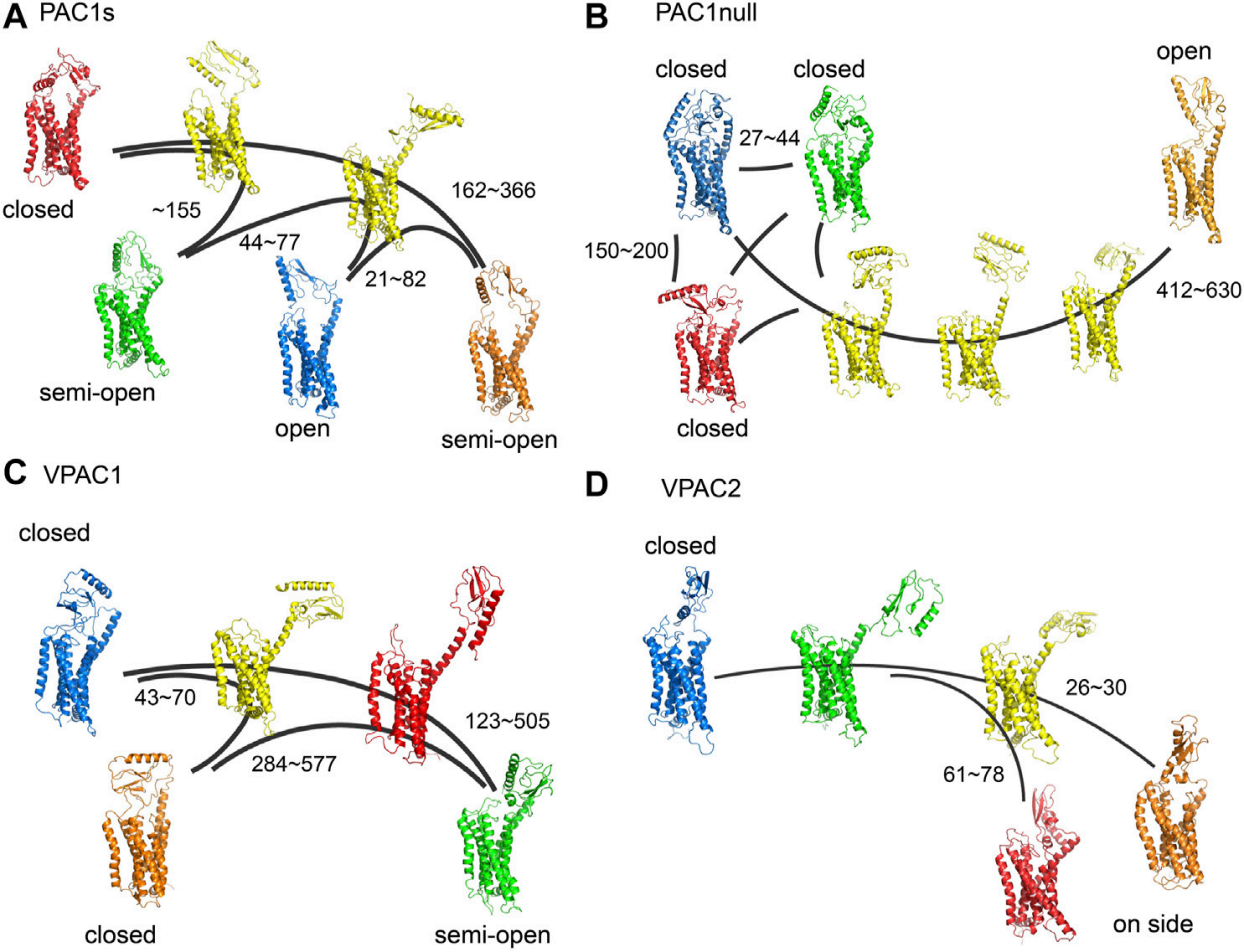
**(A)** Transition pathways for **(A)** PAC1s, **(B)** PAC1null, **(C)** VPAC1 and **(D)** VPAC2 receptors. Stable conformational states are colored in blue, red, green, and orange respectively. Intermediate states are colored in yellow. Most intermediate states are ECD-unrestricted conformations. The VPAC1 conformation in red and the VPAC2 conformation in green also belong to the ECD-unrestricted conformational ensemble. PAC1null is remade from our recent study (Liao et al., 2017). The kinetic transition time between certain conformational states are labeled with units in nanoseconds.

Building on our previous study of PAC1null receptor (Liao et al., 2017), we first examined the conformations of full-length PAC1null, PAC1s, VPAC1, and VPAC2 receptors, as well as the transitions within the conformational ensembles, which are key in understanding the differential actions of these receptors beyond amino acid sequences. With MD simulations totaling 20 microseconds to sample different conformations, we constructed MSM transition pathways between conformational microstates for PAC1s and VPAC1/2 receptors to compare with those shown for the PAC1null receptor described previously (Liao et al., 2017) (Figure 1). Similar to the PAC1null receptor, the ligand-free PAC1s and VPAC1/2 receptors display a wide diversity of conformational states with respect to the ECD orientation (*θ)* and ECD-7TM backbone COM distance (*d*), which reflect in part the actions of the linker region that tethers the ECD to the 7TM. Free energy maps based on the conformation density are shown in Supplementary Figure S4, where the low energy regions correspond to the stable conformational states in Figure 1 are circled with the same color. The energy barriers between the stable conformational states vary from 0.5 to 3.5 kcal/mol. We define the closed (*θ >* 80 degree, *d* < 55 Å), semi-open (*θ = ∼*60 degree, *d* < 55 Å), and open states (*θ* = ∼40 degree, *d* < 55 Å) to distinguish these conformational states. ECD-unrestricted conformations refer to those with ECD not touching the 7TM (*d* > 55 Å), which include most intermediate states (colored in yellow in Figure 1), as well as some stable conformational states in systems of VPAC1 and VPAC2, e.g., the state in red in Figure 1C and the state in green in Figure 1D. Without binding to 7TM, they are capable to change to other stable states much faster. The conformational states in red and orange in Figure 1D that have the ECD-ECL1 backbone COM distance ∼10 Å shorter than ECD-ECL3 backbone COM distance are labeled as the on-side conformations.

For the PAC1null receptor, we previously showed that the 21-amino acid loop in ECD interacts with ECL3 to potentially sustain the open time of the receptor (Liao et al., 2017). Consistently in this work, with the deletion of the 21 amino acids in PAC1s, the receptor microstate transitions between the open conformational state and closed conformational states (Figure 1A) are faster by a factor of 2 (transition time up to 400 µs for PAC1s and up to 1,000 µs for PAC1null, Supplementary Table S3). The free energy map also exhibits ∼3 kcal/mol higher energy barrier between the open and closed states in PAC1null compared with it in PAC1s (Supplementary Figure S4). While the functional implications of these differences remain to be investigated, the open state of PAC1null, once formed, may be sustained longer than that for PAC1s to facilitate peptide trapping and PAC1null receptor activation. Overall, the conformational microstate transitions for the VPAC2 receptor appear the fastest (0.8–80 µs) compared to the VPAC1 (40–600 µs) or the PAC1 receptor variants (21–366 µs), in agreement with the free energy difference between states in Supplementary Figure S4. Therefore, despite significant primary amino acid sequence homology, the different PAC1 and VPAC receptors display unique intrinsic ensemble dynamics and conformational microstate transition times.

Several of the ligand-free receptor conformations resemble the peptide-bound receptor states identified in the structural solutions with respect to ECD orientation (*θ*) and ECD-7TM (*d*) or ECD-ECL backbone COM distances. For example, both the PAC1null and PAC1s receptors could adopt stable open conformations (*θ* = ∼40°, 45 < *d* < 55Å), which have been seen in the peptide-bound PAC1 (PDBID: 6M1I) and other Class B receptor structures, including the glucagon receptor (GCGR, PDBID: 5YQZ), parathyroid hormone 1 receptor (PTH1, PDBID: 6FJ3), and glucagon-like peptide-1 receptor (GLP-1, PDBID: 5VAI) with minimum RMSD of 4.2Å (Supplementary Figure S5). In addition, the “semi-open” conformations of the VPAC1 receptor are similar to the CGRP-bound calcitonin receptor-like receptor (CLR) structure (PDBID: 6E3Y, *θ* = ∼60°, *d* < 55Å) with RMSD of 5.4 Å. Moreover, the VPAC2 receptor “on-side” conformations with ECD in contact with ECL1 share similar features (*θ* < 80°, short ECD-ECL1 distance) with an antagonist-bound GCGR structure (PDBID: 5XEZ) with minimum RMSD of 4.6 Å. Beyond the relative orientation and position of ECD to 7TM, the 7TM conformations in the ligand-free receptor approximated those for the inactive GPCR structures (with lowest RMSD = 2.0–2.5Å in 7TM) than the peptide-bound active receptors (Supplementary Figures S6,S7), consistent with the actions of peptides to initiate transmembrane dynamics for receptor activation. In aggregate, these findings suggest that the PAC1 and VPAC1/2 receptors, even without peptide binding, have dynamic transitions between the open and closed conformational states, which prepare them for ligand binding and subsequent receptor activation/deactivation.

### Distinct Affinity of PACAP and VIP to the Receptor ECDs

**FIGURE 2:**
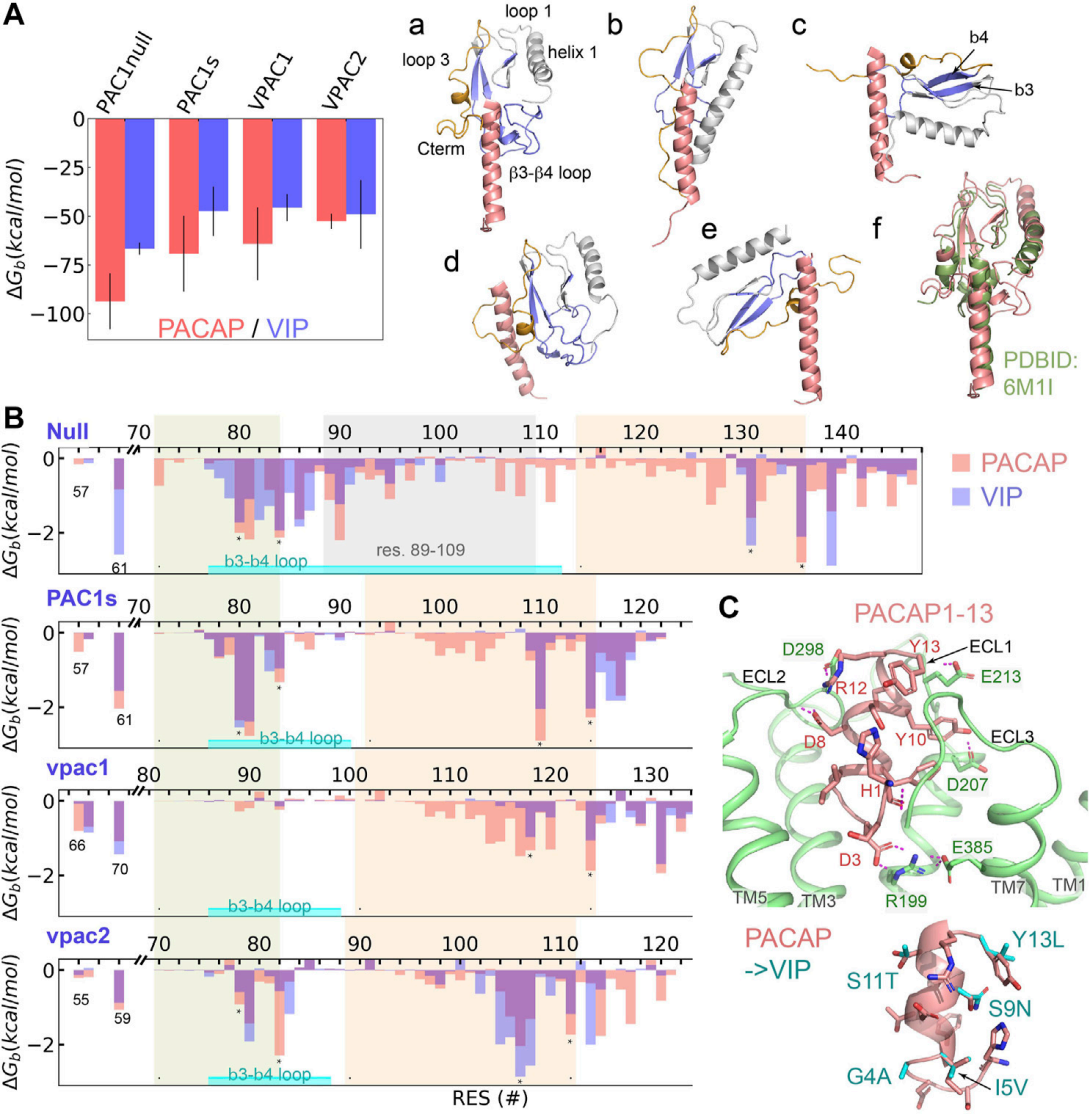
Binding selectivity and recognition of PACAP and VIP on PAC1null, PAC1s, VPAC1 and VPAC2 receptors. **(A)** Binding free energy (ΔG_b_) of PACAP and VIP on PAC1null, PAC1s, VPAC1 and VPAC2 receptors respectively by averaging the ΔG_b_ values from five replicas with bars representing the standard deviation. Representative binding conformations are displayed on the right, where residues to calculate free energy decomposition in **(B)** are colored in blue; only residues 1–27 are displayed for PACAP and VIP. **(B)** ECD per-residue free energy decomposition (average of five replicas) in PAC1 and VPAC1/2 binding PACAP (red column) and VIP (blue column). β3–β4 including the loop between is labeled. The 21 amino acids (residues 89–109) are labeled in PAC1null. **(C)** Close view of the N-terminal residues 1–13 of PACAP interacting with transmembrane orthosteric pocket of PAC1 receptor. Hydrogen bonds are shown in dashed lines. Comparison of PACAP with VIP at residues 1–13 by direct mutations is shown at the bottom right.

For Class B receptors, the ECD acts as a high-affinity peptide trap and subsequent peptide-ECD dynamics result in the presentation of the peptide N-terminus to the orthosteric binding site in the 7TM to initiate receptor activation. Our MSM analyses suggest the dynamic processes (peptide binding, large-scale ECD rotation, and peptide insertion) occur on a time scale greater than tens of microseconds. Thus, we applied a total of 40 microseconds MD simulations on the ligand-ECD systems; with the ECD-peptide complex trajectories we employed the MM-GBSA method (Miller et al., 2012) to estimate the binding free energy (ΔG_b_) of PACAP or VIP to the ECD of PAC1null, PAC1s, VPAC1 and VPAC2 receptors, as well as the ΔG_b_ contribution per residue. Our results (Figure 2A) show that PACAP binding to PAC1null receptor is the most favorable among receptor and peptide combinations. Specifically, ΔG_b_ of PACAP is around 1.4 times of ΔG_b_ of VIP in binding PAC1null ECD; the difference decreases in PAC1s and VPAC1, and become comparable for PACAP and VIP binding VPAC2 ECD (Figure 2A). This provides an energetic basis for PAC1 receptor binding selectivity for PACAP (*K*_d_ ≈ 0.5 nM) over VIP (*K*_d_ >500 nM) (Vaudry et al., 2009), which corresponds to that PACAP-binding free energy (−13.2 kcal/mol with *K*_d_ converted by ΔG_b_ = *R*TlnK_d_, T = 310 K) is about 1.5 times of VIP (−8.9 kcal/mol) in binding PAC1null, while VPAC1 and VPAC2 receptors appear to exhibit comparable binding affinity (*K*_d_ ≈1 nM) for PACAP and VIP (Vaudry et al., 2009). Notably, the experimental binding energies were obtained with full-length receptor systems, and the interactions between the ligand and 7TM may also affect the magnitude of the ΔG_b_ values for comparison with our calculation estimations. Therefore, here we compare the relative values of ΔG_b_ for the different systems as opposed to the absolute magnitude which differs from prior experiments.

To examine the ligand-ECD complex conformations, we performed structural clustering on the last 200- ns trajectories and identified six complex conformational states based on ECD orientation and the bound peptide in a vertical pose for receptor activation (Figure 2A; a-e). State (a) is found in PACAP binding PAC1null, PAC1s and VPAC2 receptors, which was resolved in the PAC1 receptor cyro-EM structure (PDBID: 6M1I, Figure 2 state(f)). State (b) in which the ECD tile angle is decreased compared to state (a) is found prevalently in PACAP/VIP binding the VPAC1 receptor. While the PAC1s receptor adopts state (a) upon PACAP binding, it establishes state (c) if bound to VIP. Similarly, the VPAC2 receptor adopts state (a) in PACAP binding, but it alters to state (e) with VIP, which is comparable to the bound ligand bound CRF1 conformation (PDBID: 2L27).

According to previous theoretical and experimental findings (Vaudry et al., 2009; Wang et al., 2020), we examined the interacting residues between the receptor ECD (Figure 2B) and the C-terminal region of PACAP/VIP (amino acid residues 13–26). The PACAP and VIP C-terminal hydrophobic V^19^xxY^22^LxxV/I^26^ sequence appears to contribute to the hydrophobic interactions with PAC1null/PAC1s and VPAC1/2 receptor residues I61^PAC^/L70^VPAC1^/I59^VPAC2^; the PACAP/VIP R^14^K^15^ residues contribute to hydrogen bonding with the ECD (Supplementary Figure S10). The β3-β4 loop in both PAC1null/PAC1s and VPAC2 receptor (environs L^80^FxxF^84,PAC^ and V^78^FxxF^82,VPAC2^ respectively) interacts with PACAP/VIP (Figure 2, state (a)). In contrast, the ECD helix 1 rather than the β3-β4 loop in VPAC1 receptor interacts with the peptide (Figure 2, state (b)). In the different receptor ECD C-terminal region, segments F^131^xxxxF^136,PAC1null^, F^110^xxxxF^115,PAC1s^, Y^118^xxxxxL^124,VPAC1^, and V^106^xxxxY^111,VPAC2^ also contribute to ligand-receptor hydrophobic interactions.

For the PAC1null receptor, there are a few notable differences in PACAP and VIP binding. Firstly, both states (a) and (d) in Figure 2 are identified in PAC1null-PACAP binding; state (a) is similar to the cyro-EM structure (Wang et al., 2020) and state (d) is close to the Nuclear Magnetic Resonance (NMR) model (Sun et al., 2007). PAC1null receptor VIP binding can also be in state (a). Secondly, PAC1null receptor hydrophobic residues 72–140^PAC1null^ play major role in PACAP binding, such as M^72^, L^80^FxIF^84^xP^86^, VW^90^, I^95^, L^106^xLxxM^111^, F^127^, F^131^, F^136^, and Y^139^ (Figure 2B); only E^142^ and D^145^ at the ECD C-terminal region contributes significantly to hydrogen bonding with the ligand. Different from PACAP, VIP can also interact with charged/polar residues 78–87^PAC1null^ such as E^79^, R^82^, N^85^ and D^87^. Lastly, PACAP interacts with residues 92–130^PAC1null^ extensively, while VIP interacts with few. This also supports the finding that another short variant with deletion of residues 53–109^PAC1null^ significantly reduces VIP binding affinity over PACAP (Dautzenberg et al., 1999). These remarkable interaction differences together contribute to the binding preference for PACAP. Compared with PAC1null, PAC1s missing the 21-amino acid loop loses the interaction formed by residues 86–111 of PAC1null which results in decrease in ligand-ECD binding affinity.

Integrating our finding on ligand-ECD interactions, PAC1 receptor cyro-EM structure (Wang et al., 2020) and biochemical data, the C-terminal region of PACAP/VIP plays a major role in binding with ECD, while the peptide N-terminus interacts with the receptor ECLs and 7TM activation site. Based on alignment with the cyro-EM structure (Wang et al., 2020), we simulated PACAP_1-13_ insertion in PAC1 7TM, without PACAP C-terminal residues 13–26 associations to the ECD. Within a few hundred nanoseconds, PACAP residues 1–4 rearranges to form a D3^PACAP^-R199^PAC1^ interacting pair (Figure 2C). The depth of peptide N-terminus into the 7TM is not significantly changed, but H1^PACAP^ reorients to form hydrogen bond with ECL3, while PACAP residues D8, Y10, R12 and Y13 interact with ECLs (Figure 2C). These interactions result in outward conformational transition of TM6 at the intracellular face of receptor. However, in longer simulations, PACAP1-13 is also observed to shift away from its primary position toward the V-gap between TM5 and TM6, allowing TM6 to transition back to former inactive conformational state. Thus, these observations show the importance of PACAP C-terminus-ECD binding to constraint the peptide N-terminus within the 7TM orthosteric interaction site and sustain the activation process.

### PACAP-Induced Activation of PAC1null Receptor

To further understand how PACAP interacts with the full-length receptor, we simulated the PAC1null receptor (a homology model of the inactive state (Siu et al., 2013; Liao et al., 2017)) bound to the PACAP_1-38_ peptide, with the first 13 residues partially inserted into the receptor 7TM. We use the Wootten numbering (Wootten et al., 2013) in this section to better demonstrate the residue positions in PAC1null receptor. Given the timescale of PAC1R activation, we combined microsecond scale MD simulation and adaptive tempering (Zhang and Ma, 2010) to enhance conformational sampling under these conditions. The N-terminus of PACAP underwent conformational rearrangement in the orthosteric site with a clear TM6 outward movement of ∼ 4 Å with the TM6-TM3 distance close to the relaxed GCGR and GLP-1 receptor structures (Supplementary Figures S11,S12), indicating an “active” state.

**FIGURE 3:**
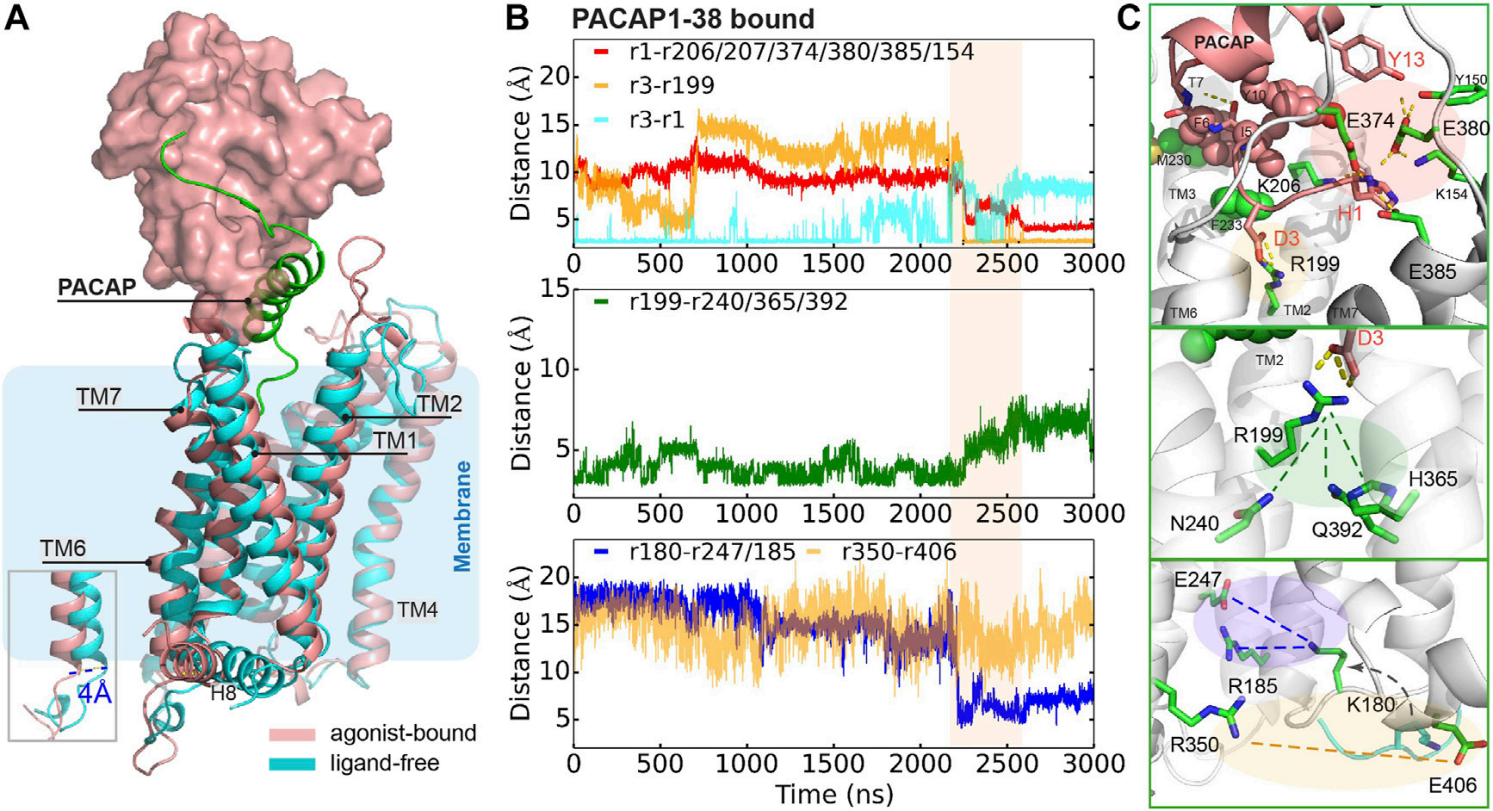
Representative conformation of PACAP-activated PAC1null receptor. **(A)** Structural alignment of PACAP-activated (red) and ligand-free (cyan) conformations. Ligand-free conformation is from our recent study(Liao et al., 2017) with only the 7TM shown. PACAP is shown in green cartoon and ECD is shown in surface representation. **(B)** Time evolution of average distance or pair distance of key charged/polar residues locating at the extracellular side, middle transmembrane, and intracellular side of the PAC1null receptor, which are displayed in **(C)** accordingly. Adaptive tempering period is in light orange strip background.

**FIGURE 4:**
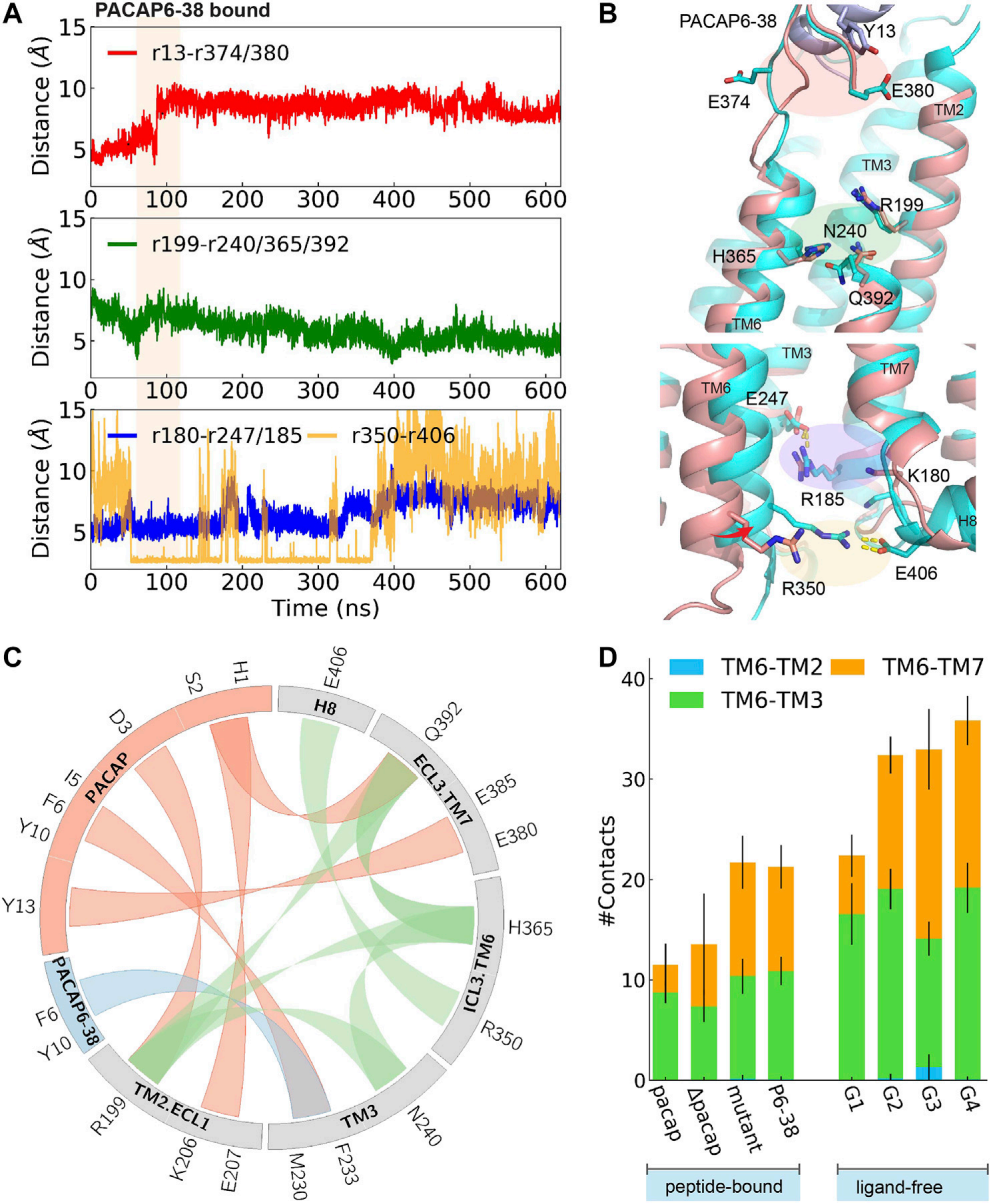
Time evolution of average distance or pair distance of key charged/polar residues locating at extracellular side, middle transmembrane, and intracellular side of PAC1null binding PACAP6-38, which are illustrated in **(B)** accordingly. Adaptive tempering period is in light orange strip background. **(B)** Final snapshot of PACAP6-38 bound PAC1null (cyan) superposed on PACAP-activated PAC1null structure (red), in which an inward shifting of TM6 was observed. **(C)** Wheel plot of major polar and hydrophobic interactions in the N-terminus of PACAP bound PAC1null (in red), N-terminus of PACAP6-38 bound PAC1null (in blue), and residue interactions within ligand-free PAC1null (in green). **(D)** Stack histogram of number of contacts between the intracellular half of TM6 (residues 344–360) and TM2, TM3, and TM7 in PAC1null receptor with: PACAP, PACAP deleted (ΔPACAP), PACAP6-38 (P6-38), and PACAP mutant (G4A, I5V, S9N, S11T, Y13L). Collections of the last 120 ns of each replica were used for the contact calculations for the PACAP, ΔPACAP, P6-38, and PACAP mutant systems. G1-G4 represent the four major ligand-free conformational states in Figure 1B. We used the last 960 ns of each 2- μs ligand-free system (Liao et al., 2017) for the contact calculations.

A detailed examination suggests that the formation of this state results from several key interactions. As shown in Figure 3B (top panel), D3^PACAP^ and PAC1null R199^2.60^ near the receptor extracellular face appears to migrate closer together in the first 700 ns, but becomes entrapped in a separate conformation for another 1500 ns. During adaptive tempering, the average distance between H1^PACAP^ and the surrounding receptor K206/D207^ECL1^, E374/E380/E385^ECL3^, and K154^1.40^ decreases gradually and appears to form hydrogen bond (H-bond) networks with an average small distance of ∼5 Å. The hydrogen bonding between the D3^PACAP^ sidechain and H1^PACAP^ amino terminus weakens during adaptive tempering resulting in the ability for D3^PACAP^ to form a new pairing with receptor R199^2.60^. As a result of D3^PACAP^-R199^2.60^ interactions, the average distance between R199^2.60^ and N240^3.43^, H365^6.52^ and Q392^7.49^ increases significantly (Figure 3B, middle panel), indicating weakened contacts between TM2, TM3, TM6, and TM7 (Figure 4D). At the receptor intracellular face, K180^ICL1^ transitions from a solvent orientation to face E247/R185, and decreasing K180^ICL1^-E247^3.50^/R185^ICL1^ distance (Figure 3B, bottom panel) converts the PAC1null receptor from an apparent “inactive” to “active” conformation that allows G protein interactions. Direct mutagenesis on residues 4–13^PACAP^ (i.e., N-terminal G4A, I5V, S9N, S11T, Y13L found in VIP sequence) in the “active” conformation caused the revocation of the spreading H-bond network around H1 and ECLs and led to inward movement of TM6 (Supplementary Figure S12) and stronger TM2, TM3, TM6 and TM7 interactions (Figure 4D).

To investigate the effects of the first few residues at the N-terminal, we removed PACAP residues 1-5 from the PACAP-bound PAC1null complex, to simulate the effects of the PAC1 receptor antagonist PACAP6-38. Starting from an active conformation, we employed 45 ns adaptive tempering to accelerate the process, as conventional MD simulations show that longer times may be needed to relax an “active” conformational state (Supplementary Figure 12B). As shown in Figures 4A,B a 4∼6 Å inward movement of TM6 was observed, indicating the closing of the intracellular G protein binding site into an inactive state. Compared to the “active” conformation, E374^ECL3^ is released and shifted to face the solvent. A return of enhanced TM2, TM3, TM6 and TM7 interactions are subsequently observed (Figure 4D), resulting in decreased average distance between R199^2.60^ and N240^3.43^, H365^6.52^ and Q392^7.49^, and the distance between the R350^ICL3^-E406^H8^ pair (Figure 4A).

Key interactions between the N-terminus of PACAP or PACAP6-38 and PAC1null receptor is summarized in Figure 4C comparing with residue interactions in the ligand-free conformations from our previous study (Liao et al., 2017). Compared with the recent PAC1 receptor cryo-EM structure (Wang et al., 2020), key contact like D3^PACAP^-R199^2.60^ was also found in our simulations, although the N-terminus of PACAP underwent conformational rearrangements in our simulations. Together, the C-term^ligand^-ECD and N-term^ligand^-7TM interactions present the unique structure features of class B GPCR in activation: ligand-ECD binding initially constraints the peptide close to 7TM; under optimized binding state, interactions between residues 8–15 of PACAP/VIP and ECLs help dock the N-terminus of the peptide into the pocket and initiate activation (Schäfer et al., 1999).

## Conclusion

In summary, we have used advanced simulation and modeling techniques combined with prior experimental knowledge to examine the differential binding selectivity, conformation ensembles and microstate dynamic transitions among the PACAP/VIP receptor variants and subtypes, and a few key interactions in the orthosteric-binding pocket. The PAC1null, PAC1s, VPAC1 and VPAC2 receptors demonstrate unique conformation and transition states. Expectedly, from residue interactions and free energy calculations, PACAP C-terminal binding to PAC1null receptor ECD is favored over VIP. The PAC1s receptor variant harboring a 21-amino acid ECD deletion has been enigmatic because after identification from cloning, the variant appears absent or expressed at very low levels in all tissues examined to date; the PAC1null receptor by contrast is dominant. Our analyses show that the ECD 21-amino acid loop in the PAC1null receptor allows several functional advantages including potential enhancement of the receptor open state to facilitate peptide entrapment and increasing peptide binding affinity. The simulations reveal the receptor conformational changes upon ECD-bound peptide dynamics and peptide N-terminal presentation to the 7TM orthosteric activation site. Notably, the interaction of PACAP residues 8–15 to the ECLs is observed to help dock the peptide N-terminal into the orthosteric pocket and facilitate D3^PACAP^–R199^PAC1null^ interactions that result in 7TM transitions and TM6 dynamics to initiate activation. Interestingly, the ECD not only functions for high affinity binding but also appears to constraint the orientation of the peptide N-terminus in the orthosteric site to maintain the activation state. These observations may be common to other Class B GPCRs and provide mechanistic insights for the development of therapeutics.

## Data Availability

The original contributions presented in the study are included in the article/Supplementary Material, further inquiries can be directed to the corresponding author.
