# Supplementary material for: Molecular Basis of Class B GPCR Selectivity for the Neuropeptides PACAP and VIP
Source: Front Mol Biosci. 2021 Mar 25;8:644644. doi: 10.3389/fmolb.2021.644644 (PMC8027070; doi:10.3389/fmolb.2021.644644)
Supplement: Supplementary file 1 [file datasheet1.pdf]

Supplementary Material

1 Supplementary Figures and Tables

Supplementary Table 1 A summary of simulations.

Systems	Box size	No. of atoms	MD Simulation length (ns)
PAC1null ECD - PACAP	72×72×72	34499	1000×5 replicas
PAC1null ECD - VIP	72×72×72	34198	1000×5 replicas
PAC1s ECD - PACAP	72×72×72	34246	1000×5 replicas
PAC1s ECD - VIP	72×72×72	34417	1000×5 replicas
VPAC1 ECD - PACAP	72×72×72	34276	1000×5 replicas
VPAC1 ECD - VIP	72×72×72	34392	1000×5 replicas
VPAC2 ECD - PACAP	72×72×72	34296	1000×5 replicas
VPAC2 ECD - VIP	72×72×72	34477	1000×5 replicas
PAC1null (full length)	95×95×127	104443	ref[1]
PAC1s (full length)	94×94×131	107651	1000×4 replicas
VPAC1 (full length)	94×94×137	114041	1000×4 replicas
VPAC2 (full length)	94×94×141	117473	1000×4 replicas
PAC1 7TM – $PACAP_{1-13}$	86×86×96	66036	1000×2 replicas
PAC1null - PACAP	90×90×132	97564	3,000 [a]
PAC1null - ΔPACAP (deletion of PACAP)	90×90×136	102408	201, 205
PAC1null - PACAP6-38	90×90×133	100044	400, 600 [a]
PAC1null - PACAP mutation (G4A, I5V, S9N, S11T, Y13L)	90×90×130	97243	102, 103
GCGR - ligand complex (5YQZ)	90×90×145	110106	100
GLP-1 - ligand complex (5VAI)	90×90×139	104711	105

[a] Combination of conventional MD and adaptive tempering simulations.

Supplementary Table 2 Templates used in homology modeling and sequence identities.

Receptor	UniProt ID	ECD template	TMD template	TMD sequence identity with	
				GCGR	PAC1
VPAC1	P32241-1	VPAC2 (PDBID: 2X57)	GCGR (PDBID: 4L6R)	42%	60%
VPAC2	P41587-1	VPAC2 (PDBID: 2X57)	GCGR (PDBID: 4L6R)	43%	61%
PAC1[a]	P41586-1	PAC1 (PDBID: 3N94)	GCGR (PDBID: 4L6R)	41%	-

[a] used in our previous study for PAC1R (Liao, C., et al., Scientific Reports, 2017. **7**(1): p. 5427).

Supplementary Table 3 Transition time range of first and second shortest pathways.

PAC1null [a]	#1-#2 [b]	#1-#3	#1-#4	#2-#3	#2-#4	#3-#4
time (μs)	27~44	23~40	412~630	21~44	650~764	284~1010
PAC1s	#1-#2	#1-#3	#1-#4	#2-#3	#2-#4	#3-#4
time (μs)	44~77	150~200	21~82	151~155	74~140	162~366
VPAC1	#1-#2	#1-#3	#1-#4	#2-#3	#2-#4	#3-#4
time (μs)	333~500	450~550	43~70	123~505	284~577	469~488
VPAC2	#1-#2	#1-#3	#1-#4	#2-#3	#2-#4	#3-#4
time (μs)	0.7~0.8	61~78	26~30	14~18	24~26	73~82

[a] from our recent study (Liao, C., et al., Scientific Reports, 2017. **7**(1): p. 5427). [b] stable states four microsecond MD simulations (#1~#4) in consistent with Supplementary Figure M7.

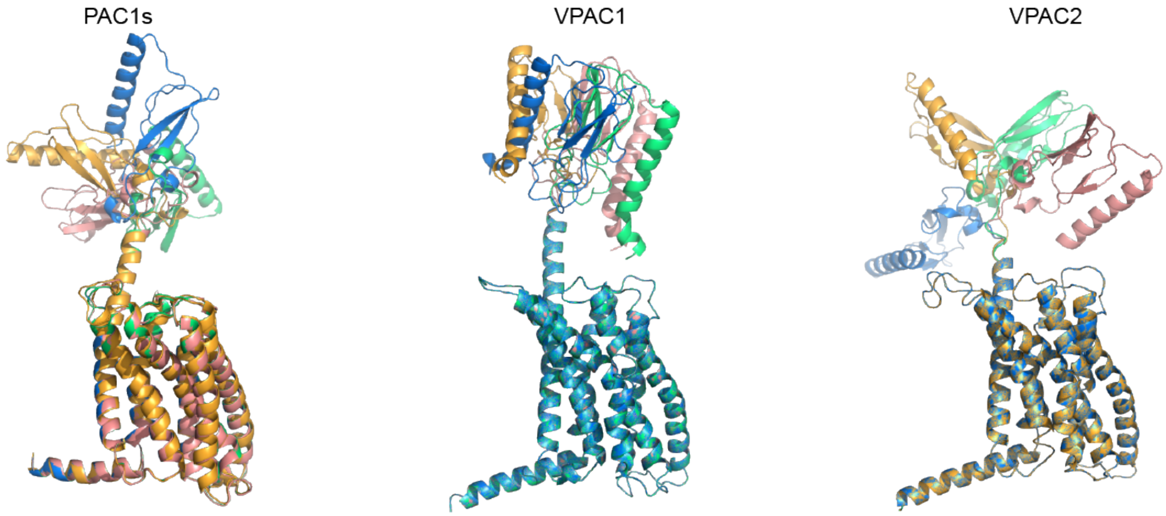

Supplementary Figure 1. Side views of initial models differing in ECD orientations by rotating the backbone dihedrals in the linker. Replicas #1, #2, #3, and #4 are colored in blue, green, red, and orange colors respectively, same color scheme as in Supplementary Figs 5 and 6.

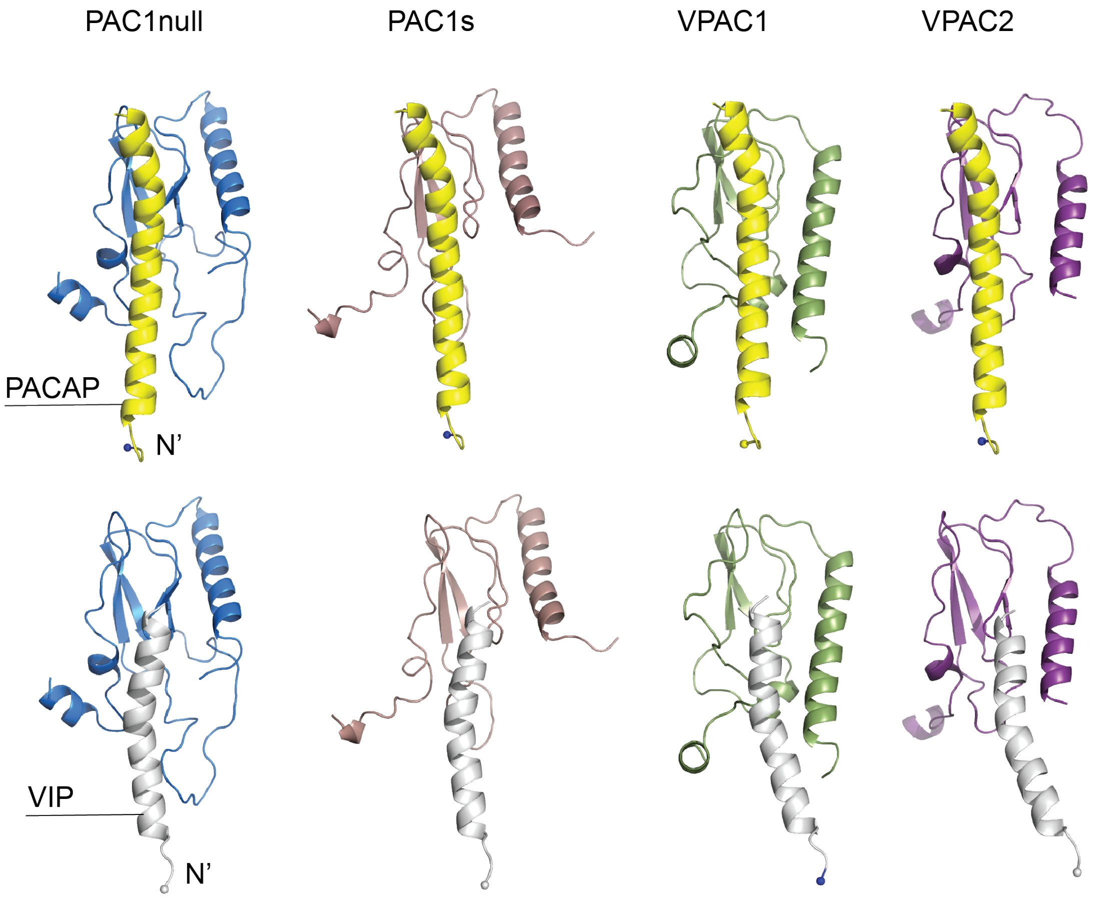

Supplementary Figure 2. Initial structures of PACAP/VIP – ECD for PAC1null, PAC1s, VPAC1 and VPAC2 systems. ECD from different receptors are shown in different colors; PACAP is in yellow and VIP is in white. The peptide was placed ~1 nm away from the ECD.

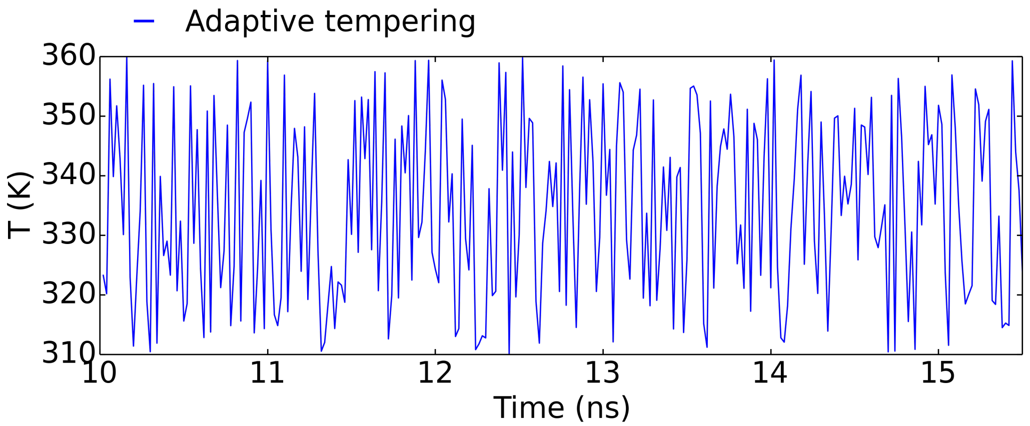

Supplementary Figure 3. Demonstration of temperature evolution in time series in our adaptive tempering simulation.

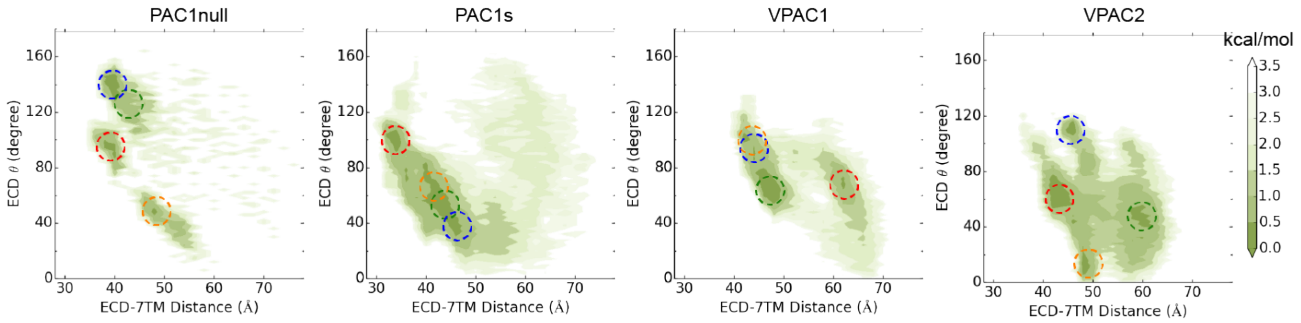

Supplementary Figure 4. Free energy maps based on conformation density of the ECD orientation (θ) against ECD-7TM center-of-mass (COM) distance from four microsecond MD simulations (#1~#4), in which the low energy regions correspond to the stable conformational states in Fig 1 are circled with the same color scheme.

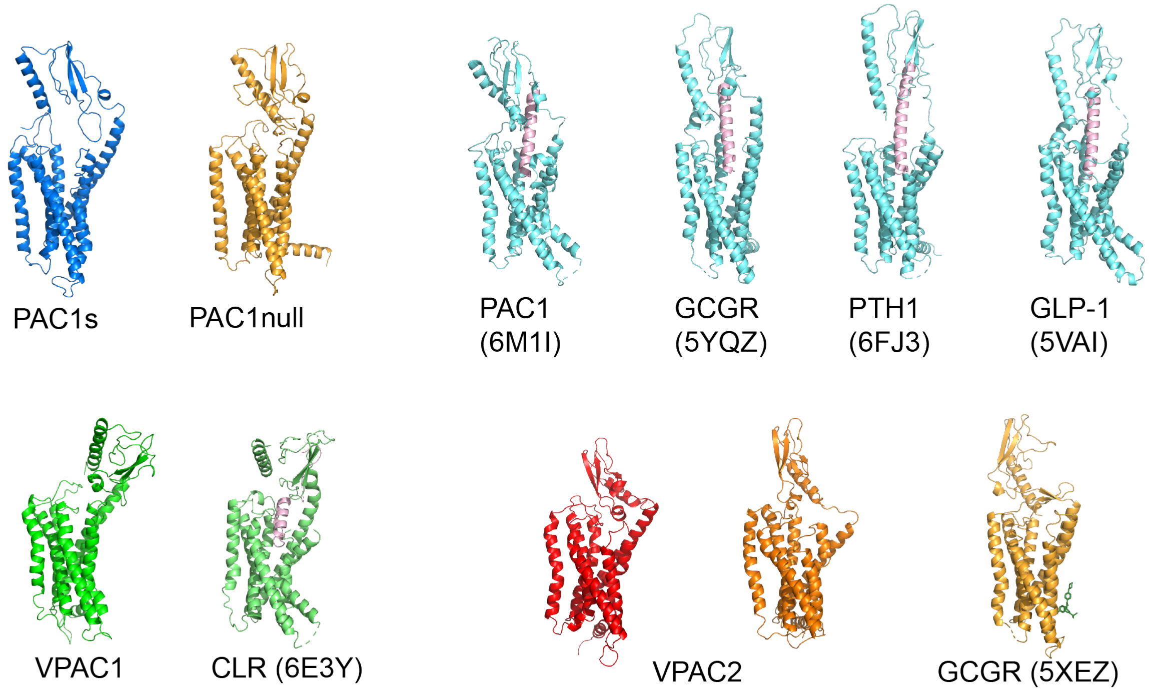

Supplementary Figure 5. Conformations sampled in our simulations that resemble the crystal or cyro-EM structures of class B GPCR (with PDBID in brackets) with respect to ECD orientation, $d_{\mathrm{ECD\text{-}ECL1}}$, and $d_{\mathrm{ECD\text{-}ECL3}}$. PAC1null receptor is from our recent study [1].

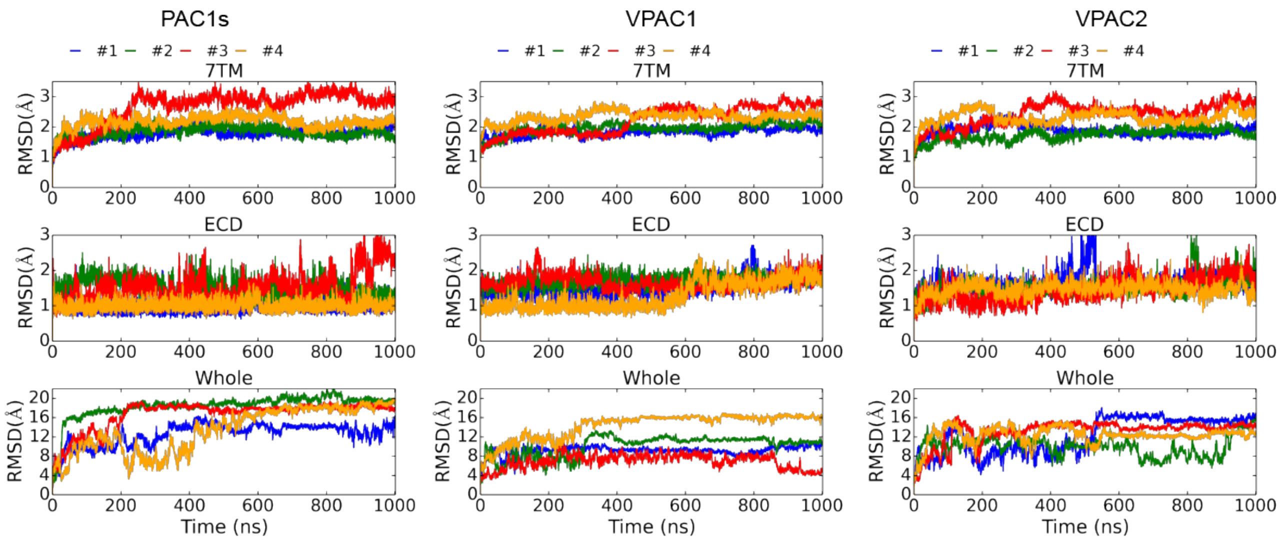

Supplementary Figure 6. Time evolution of C_α RMSDs of ECD, 7TM, and the entire protein. RMSDs were computed by alpha carbon alignments on initial structures. Each reaches a relatively stable conformation.

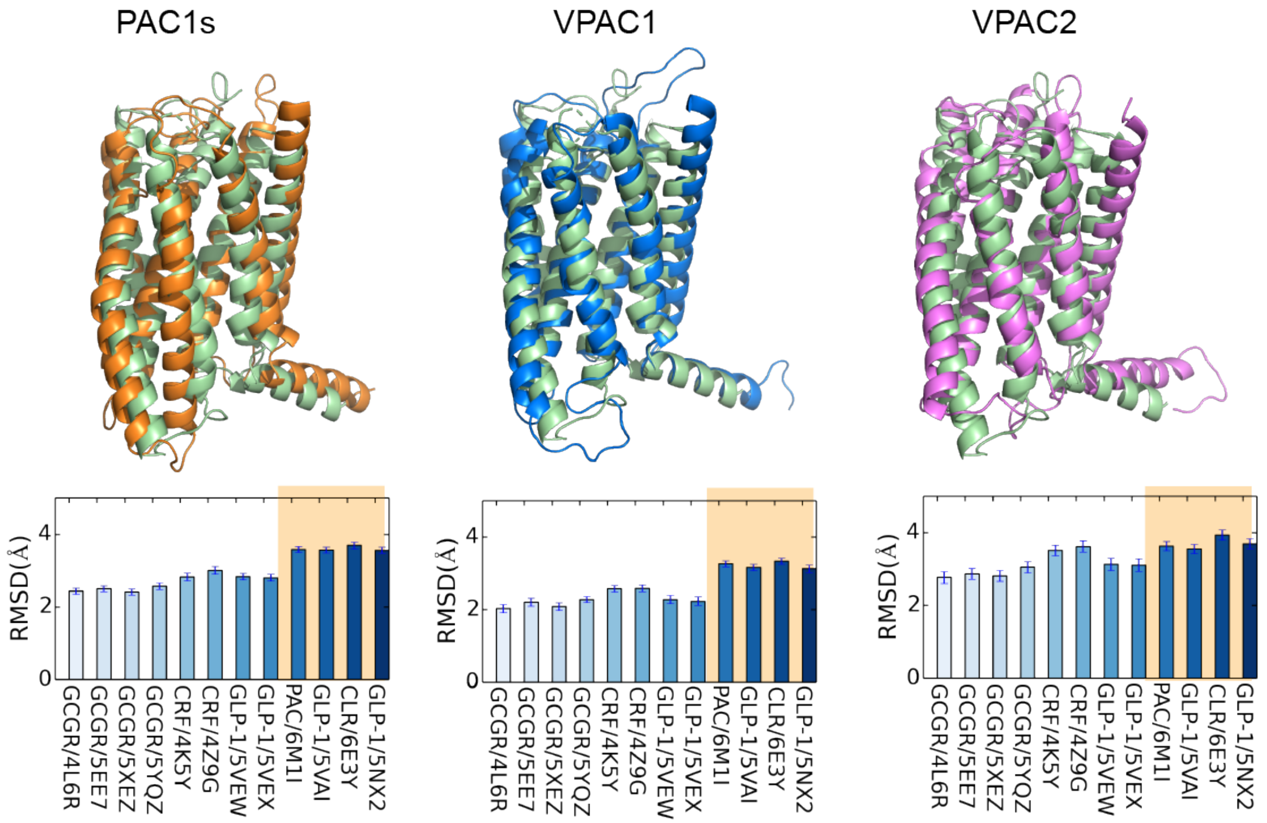

Supplementary Figure 7. Representatives for PAC1s, VPAC1 and VPAC2 superposed with GCGR (PDBID: 5EE7). Bottom: RMSDs with standard errors computed by 7TM alignments on series of class B GPCR structures: GCGR, CRF1, CLP-1, PAC1, and CLR structures (names followed by PDBIDs). The last four columns (highlight in light orange) are proteins structures in active states.

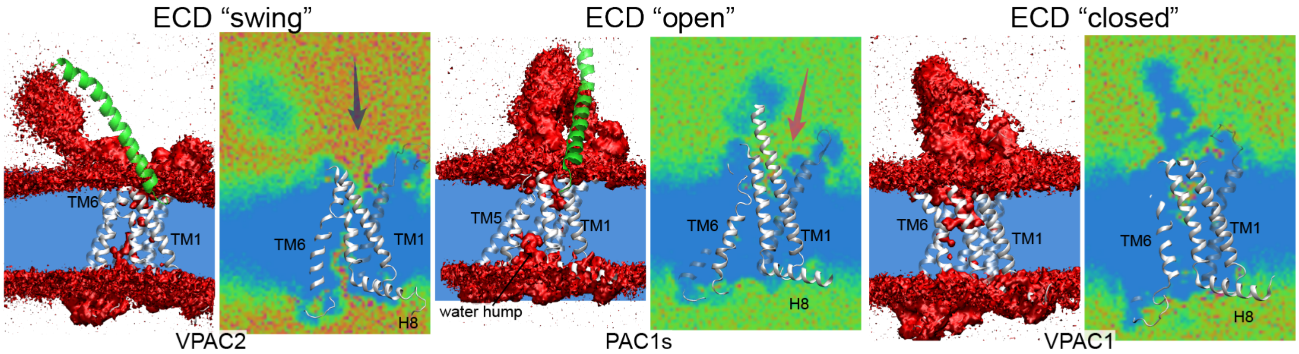

Supplementary Figure 8. Water density around ECD "swing", "open" and "closed" conformations with section views indicating the accessibility of PACAP/VIP to the orthostatic pocket in PAC1 and VPAC1/2. The ECD "open" and "swing" states exhibit larger water regions near the upper cavity, while the "closed" conformations which largely cover the orthostatic pocket with ECD have less water filled region. The open areas of the orthostatic site in ECD open and swing conformations have suggested that when a potential ligand (in green) binds to the ECD, it is able to dock in the orthostatic pocket along with ECD motions, which further open TM6 with enlarged water hump at the cellular side readily for G protein binding. In general, conformation ensemble and transitions in PAC1null, PAC1s, VPAC1, and VPAC2 have displayed unique features regarding ECD motions, ligand binding, and access to the orthostatic pocket, which further affect the fine-tune of neuropeptide-induced signaling.

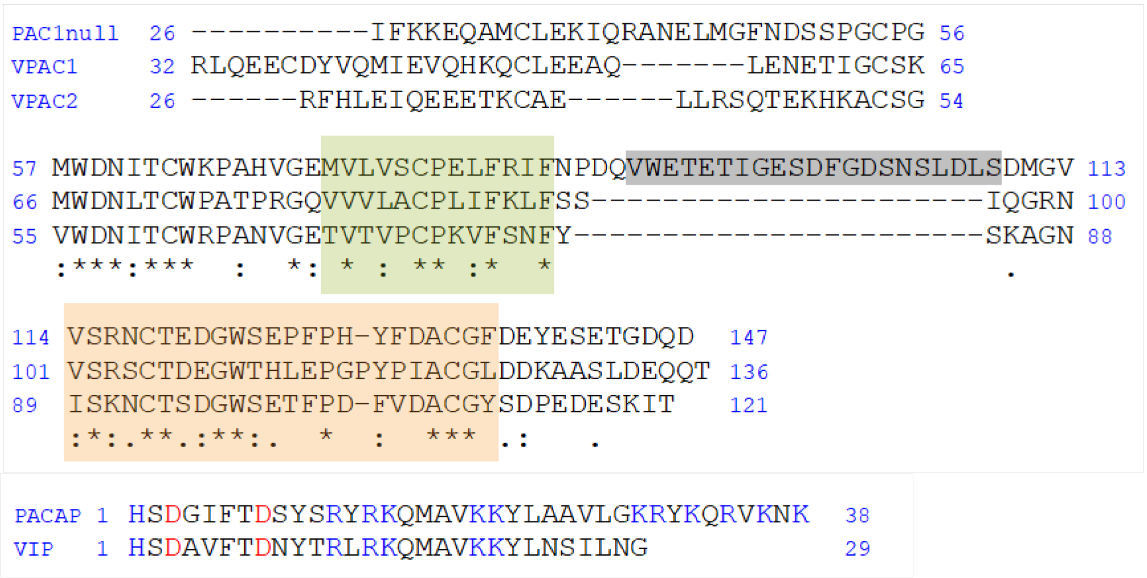

Supplementary Figure 9. Seqences of PAC1null ECD, VPAC1/2 ECD, and PACAP/VIP in this study. The 21 amino acids (res. 89-109) in PAC1null are highlighted in grey. Sequences highlighted correspond to the same color scheme in Fig 2B of the main manuscript.

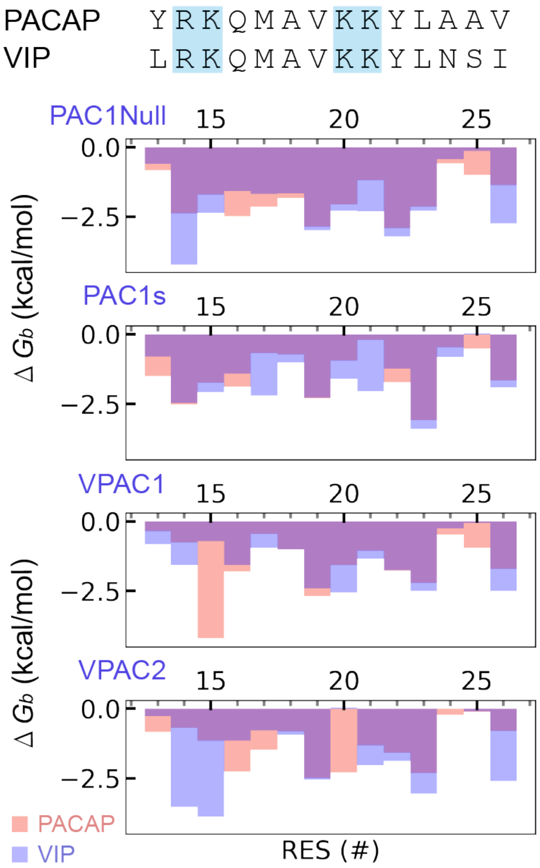

Supplementary Figure 10. Per-residue free energy decomposition of res. 13-26 of PACAP/VIP in PAC1null, PAC1s, VPAC1, and VPAC2 binding.

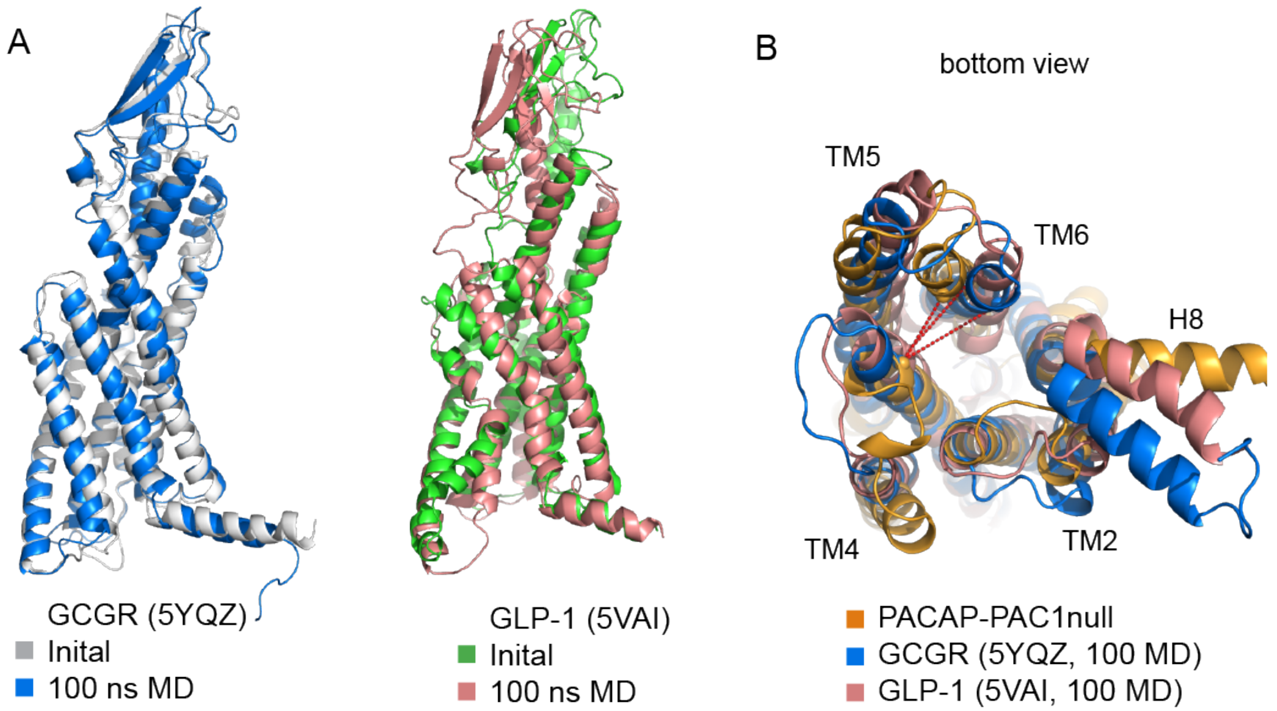

Supplementary Figure 11. (A) Initial and final structural alignments of glucagon receptor (GCGR, PDBID: 5YQZ) and GLR-1 receptor (PDBID: 5VAI) in 100-ns MD. (B) Bottom view of superposed final ligand-bound PAC1R, GCGR, GLR-1 receptor structures. Cα distance of $L251\text{-}L351_{(PAC1R)}$, $L249\text{-}L347_{(GCGR)}$, $L251\text{-}L349_{(GLP\text{-}1R)}$ representing TM6-TM3 distance in Supplementary Material Fig 11 are shown in red dash lines.

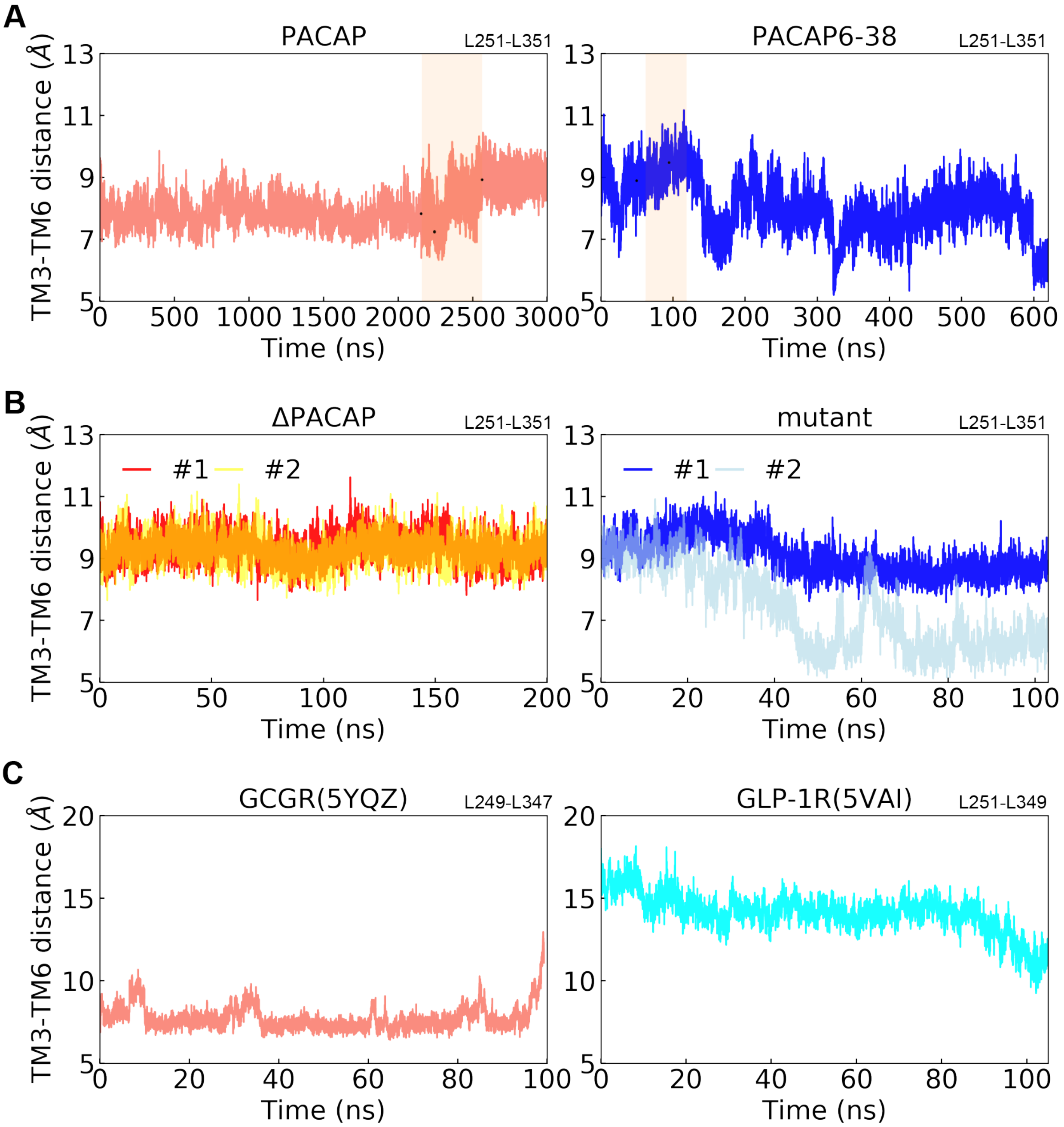

Supplementary Figure 12. (A) Time evolution of TM3-TM6 distance (by Cα distance between L251 and L351) in PACAP and PACAP6-38 bound PAC1null simulations. Adaptive tempering is in light orange strip background. (B) Time evolution of TM3-TM6 distance in Δpacap (deletion of PACAP) and PACAP mutant (G4A, I5V, S9N, S11T, Y13L) in PAC1null simulations. (C) Time evolution of TM3-TM6 distance in ligand-bound GCGR and GLP-1 receptor simulations.

2 Construction, validation, and analyses of Markov state model

Markov state models of molecular kinetics (MSMs) were applied on the collection of ligand-free MD trajectories, in which we could identify the transition pathways and estimate the long-time statistical dynamics between the conformational states of interest. We used the MSMBuilder3.8.0 program[2, 3] to construct the transition matrices of MSM and calculated the transition timescale based on the transition-path theory.[4-7] A general scheme is displayed below.

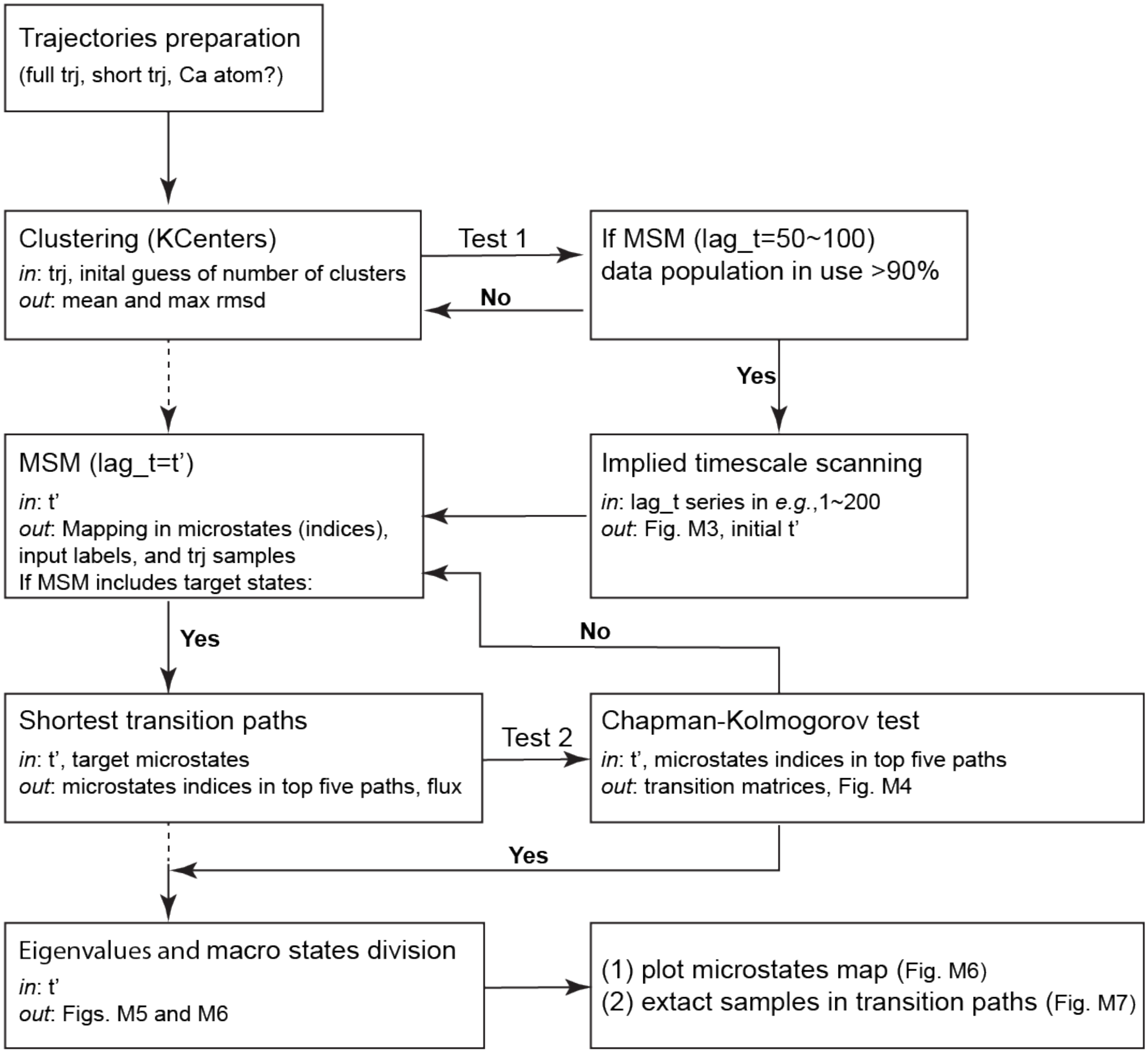

Supplementary Figure M1. General scheme for MSM constructions in present study.

(1) Trajectories preparation. We first prepared the trajectory dataset with atoms indices by saving the C_α coordinates of a GPCR protein excluding the first and last five terminal residues which are usually dynamic. We first tried to build a MSM on full trajectories (Test1 in Supplementary Fig M1), it turns out data population in use were as low as 50% since finals states take up large population in full trajectories. Therefore, we trimmed our trajectories based on RMSD plots (Supplementary Fig M2)

and visually checking the trimmed trajectories to make sure trajectories contain final conformation population and also pass the initial test. In general, we have 4,519~14,281 configurations.

(2) Clustering. We grouped the conformation dataset into a set of clusters, called microstates, based on structural similarities. We used the *k*-centers algorithm[2] to group the dataset into 33~55 clusters by RMSD metric and make sure mean distance and maximum distance were not too large, within ~0.35 Å and ~0.59 Å, respectively.

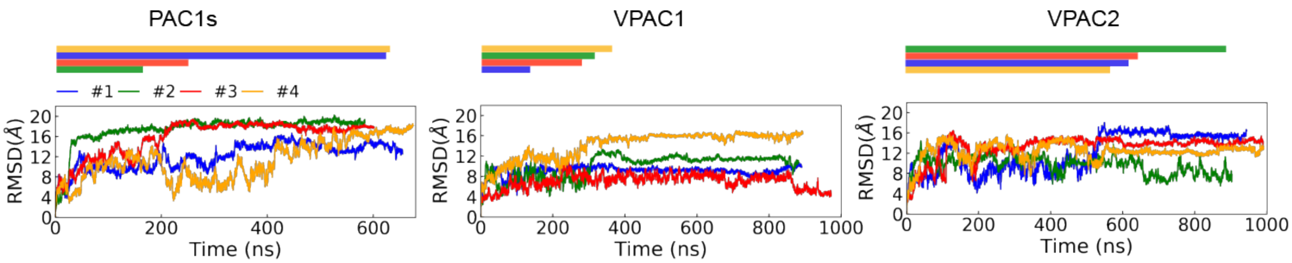

Supplementary Figure M2. Time evolution of RMSDs of the entire protein. RMSDs were computed by C_α alignments on initial structures. The lines on top indicate where the data were trimmed for MSM construction.

(3) A MSM construction. With the set of microstates discretization, a series of microstate transition matrices in the evolution of the observation interval or lag time at τ, 2τ, …, $n\tau$, were constructed, on which the implied timescales and the *Chapman-Kolmogorov* test[8] were carried out to examine if a microstate transition matrix with the microstate discretization and a chosen lag time is Markovian.[5, 8, 9] We used maximum likelihood estimation to build the transition matrices at series of lag times, in which increasing the lag time means that states can get larger and more coarse grained as the longer lag time the fewer states are kinetically relevant (kinetically reach each other on timescales faster than the lag time).[10]

(4) Implied timescales scanning. The implied timescales as a function of the lag time are shown in Supplementary Fig M3. Plots with gaps between gradually level out, from which we give initial guess for lag time (marked as t') that is Markovian. We constructed MSM with lag time = t', from which we collected these microstates (labels) that include the final conformations to calculate the short pathways connecting these stable conformational states in a transition matrix.

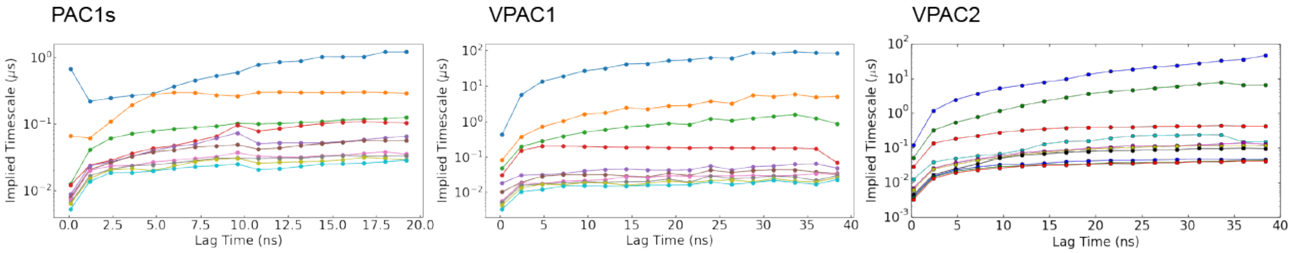

Supplementary Figure M3. Implied timescales as a function of the lag time. The macrostate partitions become robust along lag time series.

(5) Shortest transition pathways. Using the transition-path theory (TPT),[4-7] we calculated the top five shortest pathways connecting these stable conformational states in a transition matrix, each with

the minimum transition net flux in a pathway, given lag time = t'. We collected two sets of microstates that are in the shortest and second shortest pathways to go through the *Chapman-Kolmogorov* test.

(6) The *Chapman-Kolmogorov* test[5, 8] were applied on set of states that constituting the first and second shortest paths between stable conformational states as shown in Supplementary Fig M4.The detailed implementation of *Chapman-Kolmogorov* test was described in a specific chapter [11]. We tested if the lag time = t' holds the test, i.e., the transition probabilities from MSM agree well with the probabilities in observed trajectory within statistical uncertainty at lag time = t'. If not, we go back to last step with a new lag time again. If yes, we build up a macrostates MSM with lag time = t'.

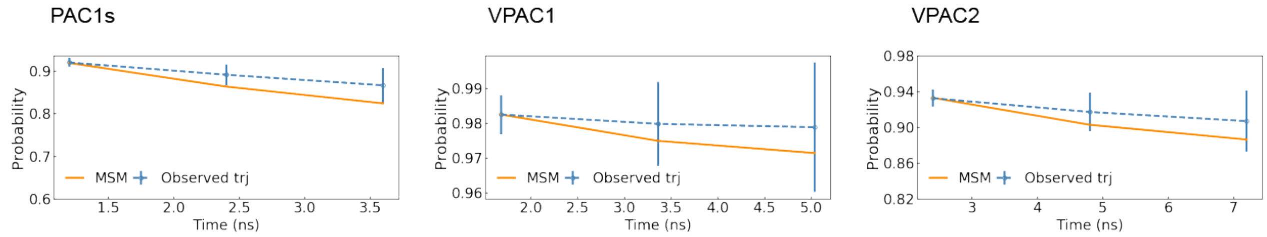

Supplementary Figure M4. Groups of microstates that constitute the shortest and second shortest transition pathways between closed and open states were examined by *Chapman-Kolmogorov* test. The transition probabilities from MSM agree well with the probabilities in the observed trajectories within statistical uncertainty.

(7) The eigenvalues of the transition matrix are given with lag time = t', shown in Supplementary Fig M5. The division of macrostates were calculated from the eigenfunction structure using the *Perron Cluster Cluster Analysis* (PCCA) method.[12]While the macrostates number can be visually estimated by the number of the major gaps in the implied timescales, it is directly determined here by the number of eigenvalues that are close to 1.[12]

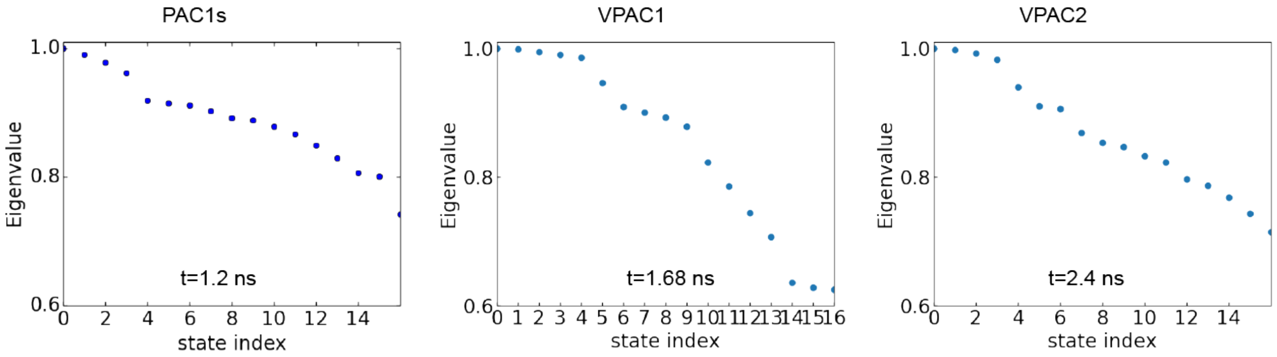

Supplementary Figure M5. Eigenvalues of the transition matrix at lag time of 1.2, 1.68, 2.4 ns for PAC1s, VPAC1 and VPAC2 systems. Only the first sixteen data are shown.

(8) We map the microstates division in the 3D orientation-distance variable space (Supplementary Fig M6) and extracted samples in transition paths displayed in Supplementary Fig M7. We obtained the time to travel from one set of states to the other by the lag time divided by the minimum transition net flux. The average transition time of the first two shortest pathways are listed in Supplementary Table 3.

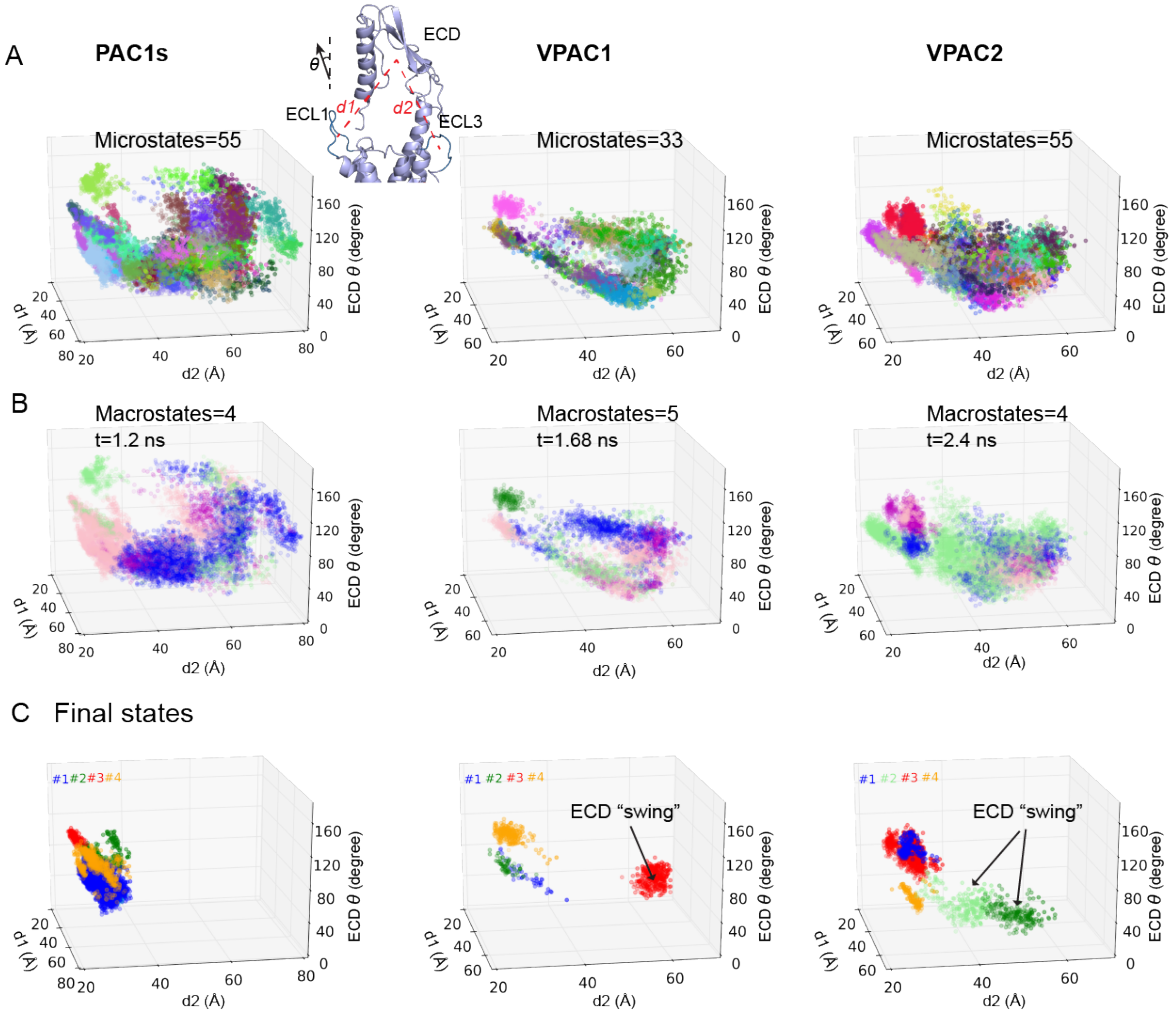

Supplementary Figure M6. Conformational state divisions in MSM in projection to ECD tilt angle (θ) and position by ECD-ECL1 (d1) and ECD-ECL3 (d2) center-of-mass distance for PAC1s, VPAC1/2R systems. (A) Divisions of microstates marked by different colors. (B) Divisions of macrostates marked in different colors at lag time of 1.2, 1.68, 2.4 ns. (C) Microstates containing final conformational states (marked as #1, #2, #3, #4) in each system.

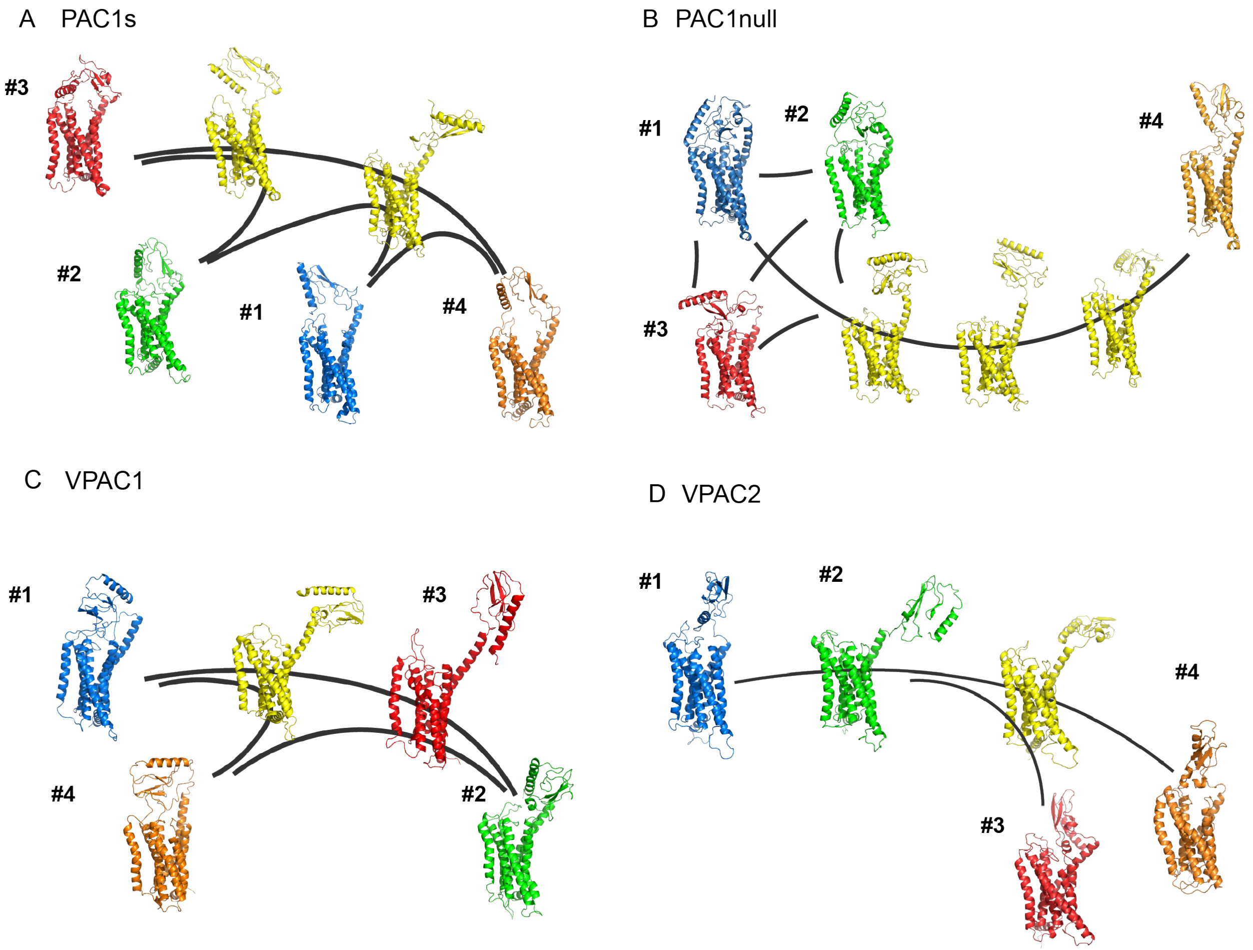

Supplementary Figure M7. Transition pathways of PAC1s, PAC1null, VPAC1 and VPAC2. Intermediate states are in yellow carton. The transition pathway of PAC1null was remade from our recent study [1].

Reference:

1. Liao, C., et al., *Conformational Transitions of the Pituitary Adenylate Cyclase-Activating Polypeptide Receptor, a Human Class B GPCR.* Scientific Reports, 2017. **7**(1): p. 5427.
2. Bowman, G.R., X.H. Huang, and V.S. Pande, *Using generalized ensemble simulations and Markov state models to identify conformational states.* Methods, 2009. **49**(2): p. 197-201.
3. Bowman, G.R., et al., *Progress and challenges in the automated construction of Markov state models for full protein systems.* J Chem Phys, 2009. **131**(12): p. 124101.
4. E, W. and E. Vanden-Eijnden, *Towards a theory of transition paths.* Journal of Statistical Physics, 2006. **123**(3): p. 503-523.
5. Noe, F., et al., *Constructing the equilibrium ensemble of folding pathways from short off-equilibrium simulations.* Proceedings of the National Academy of Sciences of the United States of America, 2009. **106**(45): p. 19011-19016.
6. Berezhkovskii, A., G. Hummer, and A. Szabo, *Reactive flux and folding pathways in network models of coarse-grained protein dynamics.* Journal of Chemical Physics, 2009. **130**(20): p. 205102.
7. Metzner, P., C. Schutte, and E. Vanden-Eijnden, *Transition Path Theory for Markov Jump Processes.* Multiscale Modeling & Simulation, 2009. **7**(3): p. 1192-1219.

8. Prinz, J.H., et al., *Markov models of molecular kinetics: Generation and validation.* Journal of Chemical Physics, 2011. **134**(17): p. 174105.
9. Chodera, J.D., et al., *Automatic discovery of metastable states for the construction of Markov models of macromolecular conformational dynamics.* Journal of Chemical Physics, 2007. **126**(15): p. 155101.
10. Pande, V.S., K. Beauchamp, and G.R. Bowman, *Everything you wanted to know about Markov State Models but were afraid to ask.* Methods, 2010. **52**(1): p. 99-105.
11. Liao, C., V. May, and J. Li, *Assessment of Conformational State Transitions of Class B GPCRs Using Molecular Dynamics.* Methods in molecular biology (Clifton, N.J.), 2019. **1947**: p. 3-19.
12. Noe, F., et al., *Hierarchical analysis of conformational dynamics in biomolecules: transition networks of metastable states.* J Chem Phys, 2007. **126**(15): p. 155102.
